# Aerial images and water quality dataset for fishpond's condition monitoring

**DOI:** 10.1016/j.dib.2023.110009

**Published:** 2023-12-23

**Authors:** Dany Eka Saputra, Dani Suandi, Joshua Wenata Sunarto, Petra Michael

**Affiliations:** Computer Science Department, School of Computer Science, Bina Nusantara University - Bandung Campus, Jakarta, Indonesia

**Keywords:** Temperature, pH level, Total dissolved solid, Sensor data, Image processing

## Abstract

This dataset is part of fundamental research to produce IoT monitoring in fishponds. The data consists of the results of measurements of pH, total dissolved solids (TDS), and water temperature obtained through manual sensor devices in several locations at different times. Additionally, this data also includes images taken by drones at consistent heights. These images are linked to the sensor data that has been collected. In this research, data will be used to monitor the health of fishponds through visual data. This data can be used for correlation analysis between visual data and sensor data. The hypothesis is the visual appearance of the pond (the colour) is affected by the number of mixed solid (mud and other organic material) in the water, which reflected in the TDS level of the water. In addition, the data can also be used for initial investigations into the development of machine learning models for pool condition recognition through image analysis.

Specifications Table

**Table:** 

Subject	Computer Science
Specific subject area	Computer Vision and Pattern Recognition
Data format	Filtered
Type of data	.csv (dataset with label).jpg (image dataset)
Data collection	The data was collected on two locations at different time. To collect the data, multiple digital sensors was used. Temperature data was collected using DS18B20 temperature sensor. The pH level data was collected using Xingweiqiang Digital pH meter. The total dissolved solid was measured using Portable E1 TDS&EC meter. The fishpond image was taken using DJI Mini drone at consistent height, while a small number of images was taken using smartphone camera. The data was collected at morning, noon, and afternoon on several days to ensure the variance on the conditions. The data collection was conducted between May and October 2023. The data was then processed as tabular data and then uploaded to Mendeley Data [1] and marked as free to download for public.
Data source location	The data was collected on several fishponds on two different locations as follows: a. Institution: Private small freshwater fish farm; City: Tasikmalaya, West Java; Country: Indonesia; Coordinate: 7°20′53.6"S 108°11′14.2"E. b. Institution: Paskal Hypersquare; City: Bandung, West Java; Country: Indonesia; Coordinate: 6°54′51.9"S 107°35′33.4"E.
Data accessibility	Repository name: Mendeley Data Data identification number: 10.17632/rtsrk8792k.2 Direct URL to data: https://data.mendeley.com/datasets/rtsrk8792k/2">https://data.mendeley.com/datasets/rtsrk8792k/2 No access control is required to view or download the datasets

## 1 Value of the Data

• The dataset contains information on fishpond conditions, visually and numerically. The data are useful in investigation on method to determine fishpond water quality through visual means.
• Researchers may benefit from this dataset through the dataset's linkage on visual data with water quality data. The researcher may study or develop means of inferring the fishpond's condition using visual data using this dataset. It is expected that the visual condition is affected by the number of solid mixed in the water, in which reflected in the water quality parameter.
• These data can be processed using correlation analysis or clustering method, or any image processing method. The status label can be used to enhance the result of correlation analysis and clustering methods.

## 2 Data Description

The dataset comprises 1023 data entries, with each entry representing the condition and a 100×100 pixels visual image of a fishpond captured at a specific time and location. The original image file of the fishpond has also been provided as raw data. The dataset is comprised of a tabular data (in csv file), a folder of raw images (in JPG format), and a folder of cropped images (in JPG format).

The csv file encompasses tabular information from 21 distinct ponds, each corresponding to different locations and collection times. Additionally, the filename for the visual image (cropped and raw images) is provided for each data entry. Table 1 shows the attributes of the tabular information within the dataset.

**Table 1:** Fish pond monitoring dataset attribute table with images.

Attribute	Descriptions	Filtered Data Range
Pond_Label	Char	
Temp	Float	25.5 - 33.5 degrees Celsius
TDS	Integer	145-297 mg/L (ppm)
pH	Float	6.8 - 9.6
State	Char	
raw_images	Char	
images	Char	

The explanation of each attribute is as follows:

a. Pond_label. Provides the label that informs where the data entry is taken. Each pond number corresponds to a different time and location of data collection.
b. Temp. Provide the data about the temperature of water. The temperature is given in degree Celsius.
c. TDS. Provide the data on the level of total dissolved solid on the water. It is given as mg/L.
d. pH. Provide the data on the level of acidity of the water.
e. State. Provide the classification of water condition in relation to the optimal growing conditions of Oreochromis niloticus. Each entry may have several labels attached, depending on the parameters in each entry. For entry in which all parameters are within the optimal range, it only be given a label of OPTIMUM. While other conditions can be a combination of LOW_PH, HIGH_PH, LOW_TEMP, or HIGH_TEMP. The TDS in this dataset is always inside the optimal range.
f. Raw_images. Provide the filename of the raw image of the pond where the data entry is located.
g. Images. Provide the filename of the cropped image in which the data entry is located.

In relation to the state of water quality, the distribution of the label in the data entry is explained in Table 2. Each data entry may have more than one status label, so the total number of state classification in Table 2 is greater than the total numbers of data in this dataset.

**Table 2:** Number of data per state classification.

State Classification	Number of Data
LOW_PH	2
HIGH_PH	806
LOW_TEMP	191
HIGH_TEMP	43
OPTIMUM	191

The summarization of the dataset number of images and locations can be found in Table 3.

**Table 3:** Summarization of the number of images and locations.

Data Item	Number of Data
Pond's locations	21
Raw Images	105
Cropped Images	1023

## 3 Experimental Design, Materials and Methods

### 3.1 Location

The data collection was conducted on two different locations. The first location is a freshwater fish farm in Tasikmalaya Region, West Java Indonesia (7°20′53.6"S 108°11′14.2"E). The second location is a large open-air fishpond in a food court area in Bandung City, West Java, Indonesia (6°54′51.9"S 107°35′33.4"E). Data collection in both locations was conducted with written permission from the location owner.

Both locations contain similar edible fresh-water fish, mainly the *Oreochromis niloticus*. The fish in the first location are bred for consumption purposes, while in the second location only grow the fish for decoration purposes. Both locations have different water treatment methods and water sources. This condition ensures that the acquired data will have condition variance.

### 3.2 Data collection process

For each individual fishpond, the water quality data was taken on several points. These points are located on the side and the corner of the pond. In each data collection point, temperature, pH level, and total dissolved solid level were measured using digital meter and recorded. This process is repeated several different times to ensure the variance on the water quality data. The image of the fishponds on data collection time is recorded from a certain height using drone at 90° angle. There are a total of 105 images taken from all the locations.

The water quality data consists of temperature data, pH level, and total dissolved solid. These three parameters were chosen based on the research conducted by Siswanto et al. [2]. The research emphasizes these three parameters as the important parameters in supporting optimal fish growth. Each water quality data entry is given a status label that corresponds to the optimal growing conditions for *Oreochromis niloticus*. For data entry in which all parameters are inside the optimal range, the data is labeled as OPTIMUM. While for parameters that are outside the optimal range is labeled as LOW or HIGH.

### 3.3 Image data processing

**Fig. 1:**
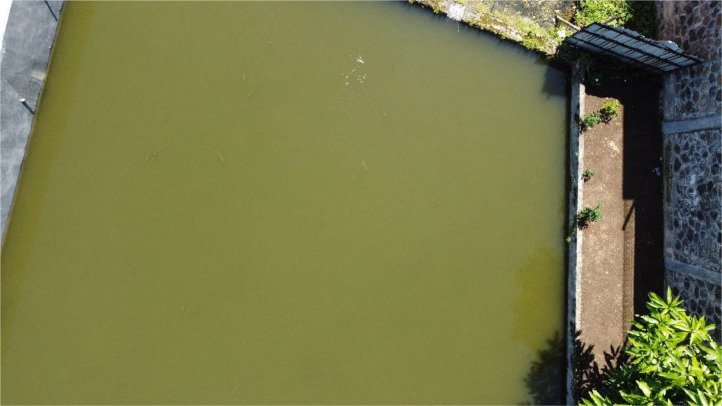
Raw image obtained from the drone.

The aerial image acquired from the drone has large resolution and includes any other objects found in the fishpond. The image needs to be clean so that only water image is used in the data. However, the raw image of each pond has also been provided in the dataset. An example of raw image from the drone can be seen in Fig. 1.

**Fig. 2:**
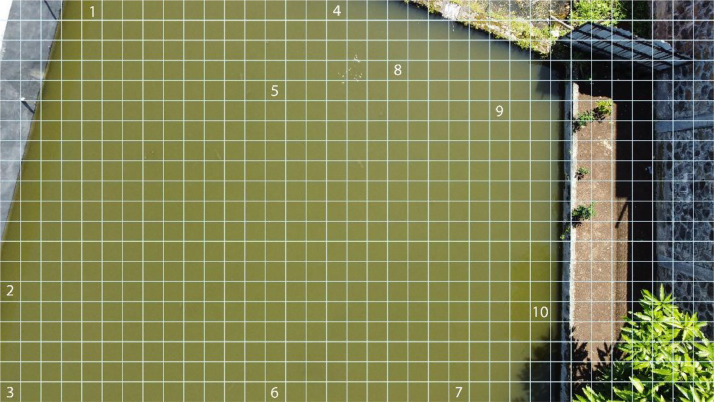
Example of overlaid gridlines and choses point.

The image was overlaid with gridlines (see Fig. 2). Each gridline was positioned so that the distance between gridlines (vertically and horizontally) is 100 pixels. So, each box formed by the gridlines has size of 100×100 pixels. On the grid, 10 boxes were chosen where it only contains the water image. Each box was then cropped to create a new image. The new image is used for the dataset. This process needs to be repeated for every raw image available in the data.

The process to layout and crop the raw image is conducted by using Python. A program is written in Python script to automatically conduct the grid layout and image cropping. The 10 data points are selected manually, to avoid any non-water image is selected. The selection was also conducted based on the proximity to the water quality data collection point. The condition of each cropped image is acquired from the result of the nearest data collection point.

## Limitations

The status label data only applies to Oreochromis niloticus growing conditions. The label may not be applicable to different species of fishes.

## Ethics Statement

The authors state to have read and follow the ethical requirements for publication in Data in Brief and confirm that the current work does not involve human subjects, animal experiments, or any data collected from social media platforms.

## CRediT authorship contribution statement

**Dany Eka Saputra:** Conceptualization, Methodology, Investigation, Supervision, Writing – review & editing. **Dani Suandi:** Data curation, Investigation, Formal analysis, Writing – original draft. **Joshua Wenata Sunarto:** Data curation, Investigation. **Petra Michael:** Data curation, Investigation.

## Data Availability

Fishpond Visual Condition Dataset v2.0 (Original data) (Mendeley Data)
